# Characteristics of the Small Bowel Lesions Detected by Capsule Endoscopy in Patients with Chronic Kidney Disease

**DOI:** 10.1155/2013/814214

**Published:** 2013-08-26

**Authors:** Harunobu Kawamura, Eiji Sakai, Hiroki Endo, Leo Taniguchi, Yasuo Hata, Akiko Ezuka, Hajime Nagase, Takaomi Kessoku, Eiji Yamada, Hidenori Ohkubo, Takuma Higrashi, Yusuke Sekino, Tomoko Koide, Hiroshi Iida, Takashi Nonaka, Hirokazu Takahashi, Masahiko Inamori, Shin Maeda, Atsushi Nakajima

**Affiliations:** ^1^Gastroenterology Division, Odawara City Hospital, 46 Hisano, Odawara 250-8558, Japan; ^2^Gastroenterology Division, Yokohama City University School of Medicine, 3-9 Fuku-ura, Kanazawa-ku, Yokohama 236-0004, Japan; ^3^Gastroenterology Division, Chigasaki City Hospital, 1-15-5 Honson, Chigasaki 253-0042, Japan; ^4^Gastroenterology Division, Yokohama Rosai Hospital, 3211 Kodukue-cho, Kohoku-ku, Yokohama 222-0036, Japan

## Abstract

Obscure gastrointestinal bleeding (OGIB) is one of the common complications in patients with chronic kidney disease (CKD), especially those who are on maintenance hemodialysis (HD). However, little is known about the characteristics of the small-bowel lesions in these patients, or of the factors that could predict the presence of such lesions. Therefore we enrolled a total of 42 CKD patients (including 19 HD patients and 23 non-HD patients), and compared the incidence of the small-bowel lesions among two groups. Furthermore, to identify predictive factors for the presence of small-bowel lesions, we performed multivariate logistic-regression-analyses. The incidence of small-bowel vascular lesions was significantly higher in CKD patients than in age-and-sex matched non-CKD patients (*P* < 0.001). On the other hand, there was any significant difference of the incidence of small-bowel lesions between HD and non-HD patients. In CKD patients, past history of blood transfusion (OR 5.66; 95% CI 1.10–29.1, *P* = 0.04) was identified as an independent predictor of the presence of vascular lesions, and history of low-dose aspirin use (OR 6.00; 95% CI 1.13–31.9, *P* = 0.04) was identified as that of erosive/ulcerated lesions. This indicated that proactive CE examination would be clinically meaningful for these patients.

## 1. Introduction

The incidence of chronic kidney disease (CKD) and the number of patients requiring maintenance hemodialysis (HD) have continued to increase in developed countries [1]. Anemia is a common feature in CKD patients [2]. It is usually normocytic and normochromic because of the decreased erythropoiesis and red blood cell survival. However, these patients can also have concomitant iron deficiency anemia (IDA) caused by gastrointestinal bleeding. Gastrointestinal bleeding is more common in CKD patients than in the general population and is also associated with a higher mortality in these patients [3]. A higher incidence of bleeding from gastroduodenal ulcers has been reported in patients with end-stage renal disease [4]. In addition, the incidence of vascular lesions such as angioectasia has also been reported to be increased in these patients [5, 6]. These findings lend support to the hypothesis that CKD patients are at a higher risk of gastrointestinal bleeding, which in turn can result in IDA. However, upper and lower gastrointestinal endoscopies often do not reveal any obvious hemorrhagic lesions in these patients.

Obscure gastrointestinal bleeding (OGIB) is defined as persistent or recurrent bleeding associated with negative findings on upper and lower gastrointestinal endoscopic evaluations [7]. It has been shown that OGIB accounts for approximately 5% of patients presenting with gastrointestinal hemorrhage [8] and that the majority of lesions responsible for OGIB are found in the small bowel [9]. The small bowel has long been a difficult organ to investigate thoroughly however, with the introduction of capsule endoscopy (CE) in 2000, noninvasive diagnosis of lesions in the small bowel has now become possible in patients presenting with OGIB [10–12]. In patients with OGIB, the diagnostic yield of CE has been found to be significantly higher than that of other diagnostic radiologic or endoscopic modalities, including push enteroscopy [13–16]; therefore, CE has come to be established as the examination modality of first choice for the investigation of OGIB.

There are a few reports investigating the incidence of small bowel lesions in CKD patients with OGIB [17, 18]; however, the sample sizes in these studies were relatively small. Moreover, little is known about factors that could predict a positive diagnosis of small intestinal lesions by CE among CKD patients. Identification of factors that might predict the presence of small bowel lesions would be clinically meaningful when considering the indications of CE. Therefore, we conducted the present study to evaluate the characteristics of small bowel lesions and to identify the predictive factors for a positive diagnosis of small bowel lesions by CE in CKD patients.

## 2. Materials and Methods

### 2.1. Patients

Of patients with OGIB who underwent CE at Yokohama City University Hospital, Odawara City Hospital, Chigasaki City Hospital, and Yokohama Rosai Hospital, between October 2007 and July 2012, a total of 42 patients with creatinine clearance values of less than 30 mL/min (CKD ≥ stage 4) were enrolled as the subjects of this study. We also enrolled 132 age- and sex-matched patients presenting with OGIB who had normal renal function (non-CKD patients) as a control group for the interpretation of the small bowel findings in CKD patients from the database at a ratio of about 1 : 3. All of the patients had undergone upper and lower endoscopic examinations recently, with negative findings. According to the bleeding pattern, OGIB was classified into two categories: overt, manifesting as melena or hematochezia, and occult, manifesting as recurrent IDA (defined as iron deficiency with blood hemoglobin <11 g/dL for women and <13 g/dL for men), and/or positive fecal occult blood test without any visible bleeding. In patients who underwent CE two or more times, only the findings of the first examination were considered for this study. Patients with failed examinations due to CE retention and/or incomplete small bowel transit were also excluded. Finally, a total of 174 patients were included for the analysis in this study.

The study protocol was approved by the Ethics Committee of each of the participating hospitals. Written informed consent was obtained from all of the subjects prior to their participation in the study.

The patient data collected included the bleeding pattern (overt or occult), age, sex, smoking history, alcohol history, blood transfusion history (within six months prior to the CE), minimum hemoglobin (Hb) level, serum creatinine, presence/absence of comorbidities (hypertension, diabetes, coronary artery disease, cerebral infarction and liver cirrhosis), and current medication history (anticoagulant drugs, antiplatelet drugs, nonsteroidal anti-inflammatory drugs (NSAIDs), histamine H2 receptor antagonists (H2-blockers), proton pump inhibitors (PPIs), and rebamipide).

### 2.2. Capsule Endoscopy

The patients were instructed to swallow the CE capsule (PillCam SB and SB 2; Given Imaging, Yoqneam, Israel) with a solution of dimethicone on the morning of the examination after overnight fasting, without any other preparation. They were allowed to drink clear liquids 2 hours after swallowing the capsule, and eat a light meal after 4 hours. Two CE experts (with experience of reporting more than 150 CE videos) separately read and interpreted the complete CE videos. If the case of a discrepancy in the interpretation occurred, the findings were reviewed simultaneously by both CE experts and a consensus was reached. In this study, capsule retention was not observed in any of the patients.

### 2.3. Definition of the CE Findings

**Figure 1:**
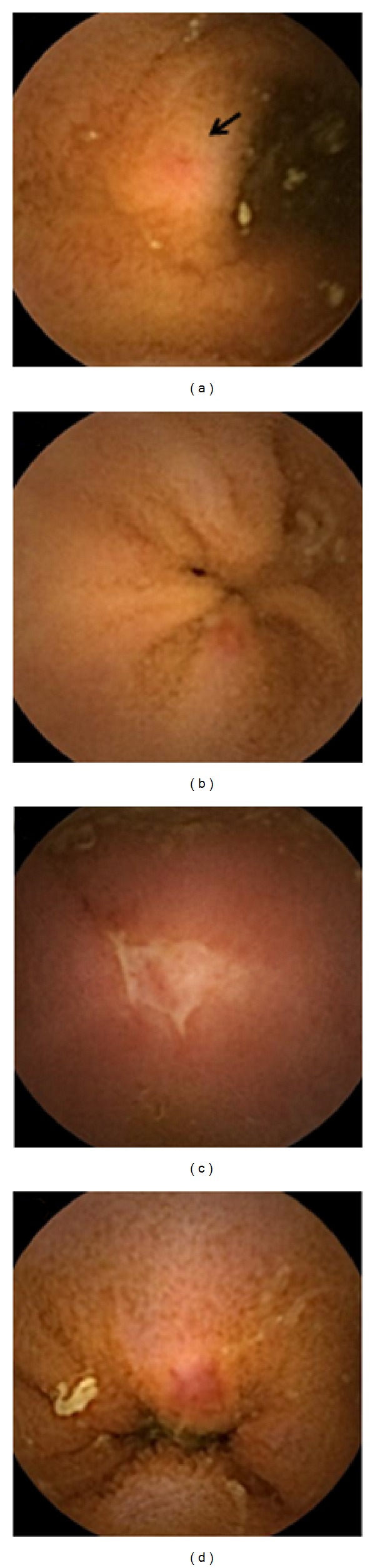
Capsule-endoscopic images of small bowel lesions. (a) Red spot representative of a less relevant (P1) lesion (black arrow). (b) Single erosion corresponding to a P1 lesion. (c) Ulceration representative of a highly relevant (P2) lesion. (d) Angioectasia corresponding to a P2 lesion.

The CE findings were classified by the P0-P2 system described in a previous report [13]. Small bowel lesions that were considered to be the cause of the OGIB, such as angioectasia, varices, arteriovenous malformations, presence of active bleeding, ulcerations, multiple (≥3) erosions, and diverticula, were classified as highly relevant (P2) lesions. Other tiny abnormalities, such as red spots, visible submucosal veins, and erosions (<3), were classified as less relevant lesions (P1 lesions). Presence of one or more highly relevant lesions was defined as a positive result of CE. Samples of the capsule-endoscopic images of small bowel lesions are shown in Figure 1.

### 2.4. Statistical Analysis

Continuous data are shown as mean ± SD. The differences in the values of the clinical parameters between the CKD and non-CKD patients were calculated by the chi-squared test and unpaired *t*-test. The differences in the incidences of the small bowel lesions between the two groups were calculated by the chi-squared test. Univariate and multivariate logistic regression analyses were performed to identify predictors of the presence of small bowel vascular lesions and erosive/ulcerated lesions in the CKD patients. Unless otherwise specified, *P* values of <0.05 were considered to denote statistical significance. All the analyses were performed using the SPSS statistical package, ver. 11.0 (SPSS Inc., Chicago, IL, USA).

## 3. Results

### 3.1. Demographic and Clinical Data

The demographic and clinical characteristics of the subjects of this study are shown in Table 1. The study patients comprised 42 CKD patients and 132 non-CKD patients ranging in age from 36 to 85 years (68.2 ± 10.5) and 38 to 86 years (68.3 ± 11.3), respectively. The number of patients with a history of recent blood transfusion was significantly higher in the CKD patient group than in the non-CKD patient group (47.6% and 28.8%, resp., *P* = 0.02). The minimum Hb concentration in the CKD patients was significantly lower than that in the non-CKD patients (7.9 ± 2.0 and 9.1 ± 2.9, resp., *P* = 0.01). Except for the history of LDA use (*P* < 0.001), concurrent use of ulcer-related medications, such as warfarin, thienopyridine, cilostazol, NSAIDs, H2-blockers, PPIs, and rebamipide, was comparable in frequency between the CKD and non-CKD patient groups (*P* > 0.05). However, the prevalence of comorbidities, including hypertension, diabetes, and coronary artery disease, differed significantly between the two groups (*P* < 0.001, *P* < 0.001, and *P* = 0.004, resp.).

### 3.2. Comparison of the CE Findings between the CKD and Non-CKD Patients

The results are shown in Table 2. The total diagnostic yield of CE was significantly higher in the CKD patients as compared to the non-CKD patients (62.0% and 43.9%, resp., *P* = 0.04). In addition, the incidence of vascular lesions was also significantly higher in the CKD patients compared to the non-CKD patients (47.6% and 20.5%, resp., *P* < 0.001). On the other hand, the incidence of erosive/ulcerated lesions was not significantly different between the two patient groups (33.3% and 27.3%, resp., *P* = 0.45).

When the differences in the diagnostic yield of CE were separately evaluated in overt and occult OGIB patients, there were no significant differences in the total diagnostic yield or the incidence of erosive/ulcerated lesions between the CKD patients and non-CKD patients in either the overt or occult OGIB group. However, the incidence of vascular lesions was significantly higher in the CKD patients compared to the non-CKD patients in both the overt (54.2% and 24.3%, resp., *P* < 0.001) and occult (38.9% and 15.5%, resp., *P* = 0.04) OGIB groups.

### 3.3. Comparison of the CE Findings between the HD and Non-HD Patients

The results are shown in Table 3. There were no significant differences in the incidences of small bowel lesions between the HD patients and non-HD patients. 

### 3.4. Factors Predicting the Presence of Small Bowel Lesions in the CKD Patients

The results are shown in Table 4. Univariate analysis identified overt bleeding (odds ratio (OR) 7.00; 95% confidence interval (CI) 1.73–28.3, *P* = 0.006), past history of blood transfusion (OR 6.22; 95% CI 1.63–23.8, *P* = 0.008), and history of alcohol drinking (OR 5.18; 95% CI 1.15–23.3, *P* = 0.03) as significant factors predictive of the presence of vascular lesions on CE. Multiple logistic regression analysis identified only past history of blood transfusion (OR 5.66; 95% CI 1.10–29.1, *P* = 0.04) as an independent factor predictive of the presence of vascular lesions in the CKD patients. On the other hand, history of LDA use (OR 6.00; 95% CI 1.13–31.9, *P* = 0.04) was identified as the only significant predictor of the presence of erosive/ulcerated lesions in these patients. 

## 4. Discussion

In this study, we evaluated the diagnostic yield of CE for small bowel lesions in CKD patients and attempted to identify predictors of the presence of vascular and erosive/ulcerated lesions in these patients. The diagnostic yield of CE in our study was 49.4%. The reported diagnostic yield of CE varies over a wide range from 40% to 60% [12, 19–23]. Therefore, the diagnostic yield in our study compared favorably with that reported in previous studies. 

The results of our study revealed a higher diagnostic yield of CE and incidence of small bowel vascular lesions in CKD patients than in non-CKD patients, which is in agreement with previous reports [17, 18]. Angioectasia is the most common vascular malformation of the gastrointestinal tract non associated with any familial syndrome or systematic lesions [24]. The etiology of angioectasia is not yet fully understood; however, previous reports have suggested intermittent submucosal venous obstruction, intermittent abnormal arterial flow, and local vascular degeneration with local hypoxaemia as potential aetiologic factors; these conditions are often observed in CKD patients [6, 25]. Therefore, CKD patients may show a higher incidence of angioectasia in the small bowel, just like the case in the stomach and colon [5, 6]. On the other hand, the incidence of small bowel erosive/ulcerated lesions was not increased in the CKD patients in our study, although some previous studies have indicated an increased incidence of gastroduodenal ulcers in CKD patients [4]. This may, in part, be ascribed to differences in the mechanisms of ulcer formation between the small bowel and gastroduodenum. An important finding of our study was that the diagnostic yield of CE in the non-HD patients was as high as that in the HD patients. This was somewhat unexpected because it would seem that systemic anticoagulation effected by intermittent heparinization in the HD patients would increase the incidence of small bowel lesions. The uremic platelet dysfunction, which contributes to an increased bleeding tendency, has been reported to occur even in pre-HD end-stage CKD patients [26], which could explain the increased incidence of small bowel lesions also observed in the non-HD CKD patients.

In this study, comorbidities such as hypertension, diabetes, and coronary artery disease were not identified as significant predictors of the presence of small bowel lesions as identified by CE, although the prevalence of these comorbidities was higher in the CKD patient group. On the other hand, past history of blood transfusion was identified as an independent predictor of the presence of vascular lesions, and history of LDA use was identified as a predictor of the presence of erosive/ulcerated lesions in the CKD patients. The occurrence of IDA in CKD patients could be explained by blood losses during HD, use of erythropoiesis-stimulating agents, and gastrointestinal bleeding. Although most patients can be managed by appropriate iron replacement, some need blood transfusion because of a progressive drop of the blood hemoglobin level [27]. Our results indicate that small bowel angioectasia may be associated with the development of IDA. LDA is widely used for primary and secondary prevention of cardiovascular disease [28], and the use of this drug is well known to be associated with an increased incidence of bleeding from gastroduodenal ulcers [29]. On the other hand, LDA has long been regarded as being safe for the gastrointestinal tract beyond the duodenum because of its rapid absorption and lack of enterohepatic circulation [30]. However, recently, an increased incidence of small bowel injury and enteropathy has been reported in patients with a history of LDA use [31, 32]. In patients with OGIB, while the bleeding often stops spontaneously, rebleeding is seen in about 40% of cases, being even life-threatening in some [33]. Specific endoscopic treatments such as argon plasma coagulation and clipping cannot only stop active bleeding, but could also decrease the risk of future rebleeding; therefore, proactive CE examination for identification of the source of OGIB in CKD patients with these risk factors is clinically meaningful.

Although our present study was a relatively large cross-sectional cohort study focusing on the characteristics of small bowel lesions in patients with CKD, it had some limitations. Firstly, our study was based on retrospective data. Although we enrolled all consecutive CKD patients with OGIB who underwent CE and selected age- and sex-matched control OGIB patients in whom the diagnostic yield of CE was consistent with most previous reports [12, 19–23], selection bias could not be avoided. Secondly, it was difficult to completely rule out all the potential causes of IDA. In addition, the anemia could have been directly associated with the CKD rather than the OGIB. Many advanced CKD patients have anemia, both associated with reduced production of erythropoietin and with iron deficiency. Therefore, it would have been difficult to prove that the anemia truly resulted from the OGIB. 

## 5. Conclusions

We demonstrated that small bowel vascular lesions were found at a higher incidence in CKD patients than in non-CKD patients. In addition, past history of blood transfusion was identified as an independent predictor of the presence of vascular lesions, and history of LDA use was identified as an independent predictor of the presence of erosive/ulcerated lesions. It is worthy of note that this tendency was observed not only in HD patients, but also in non-HD patients. Therefore, proactive CE examination would be clinically meaningful for identification of the source of OGIB in CKD patients, especially in those with a history of recent blood transfusion and/or LDA use.

## Figures and Tables

**Table 1:** Demographic and clinical characteristics of the CKD and non-CKD patients.

	CKD patients	Non-CKD patients	*P* value
Number	42	132	
Bleeding pattern			
Overt	24	74	0.90
Occult	18	58	
Demographic and behavioral characteristics			
Age, y	68.2 ± 10.5	68.3 ± 11.3	0.88
Sex, male/female	25/17	80/52	0.90
Drinking history (%)	12 (28.6)	48 (36.4)	0.35
Smoking history (%)	17 (40.5)	46 (34.8)	0.11
Blood transfusion (%)	20 (47.6)	38 (28.8)	0.02
Minimum Hb value, g/dl	7.9 ± 2.0	9.1 ± 2.9	0.01
Comorbidity, number (%)			
Hypertension	36 (85.7)	69 (52.3)	<0.001
Diabetes	18 (42.9)	21 (15.9)	<0.001
Coronary artery disease	20 (47.6)	32 (24.2)	0.004
Cerebral infarction	6 (14.3)	12 (9.1)	0.34
Liver cirrhosis	2 (4.8)	9 (6.8)	0.83
Medication history, number (%)			
Warfarin	8 (19.0)	12 (9.1)	0.08
LDA	26 (61.9)	43 (32.6)	<0.001
Thienopyridine	6 (14.3)	12 (9.1)	0.34
Cilostazol	2 (4.8)	4 (3.0)	0.59
NSAIDs	2 (4.8)	14 (10.6)	0.25
H2-blockers	13 (30.1)	36 (27.3)	0.64
PPIs	14 (33.3)	44 (33.3)	>0.99
Rebamipide	11 (26.2)	26 (19.7)	0.80

CKD: chronic kidney disease; Hb: hemoglobin; LDA: low-dose aspirin; NSAIDs: nonsteroidal anti-inflammatory drugs; H2-blockers: histamine H2 receptor antagonists; PPIs: proton pump inhibitors.

Alcohol history was defined as positive if the subject's alcohol consumption exceeded 20 g/day. Smoking history was defined as positive if the subject had smoked more than 10-pack years and was still smoking or had quit within the past 10 years. History of antiplatelet drug and/or NSAID use was defined as positive if the patient had been taking at least 1 pill per day for more than 1 week within 1 month prior to the CE. History of anticoagulant drug use was defined as positive if the patient had been taking at least 1 pill per day within one week prior to the CE.

*P* values were calculated using the chi-squared test or unpaired *t*-test.

**Table 2:** Comparison of the CE findings between the CKD and non-CKD patients.

	CKD patients (*N* = 42)	Non-CKD patients (*N* = 132)	*P* value
Abnormal CE findings (highly relevant lesions)	26 (62.0)	58 (43.9)	0.04
Vascular lesions	20 (47.6)	27 (20.5)	<0.001
Angioectasia	16 (38.1)	26 (19.7)	0.02
Arteriovenous malformations	1 (2.4)	0 (0)	—
Varices	1 (2.4)	0 (0)	—
Active bleeding with no identifiable cause	2 (4.8)	1 (0.8)	0.08
Erosive/ulcerated lesions	14 (33.3)	36 (27.3)	0.45
Ulcerations	7 (16.7)	20 (15.2)	0.59
Erosions (≥3)	7 (16.7)	16 (12.1)	0.39
Diverticula	0 (0)	1 (0.8)	—
No findings (less relevant lesions or no abnormalities)	16 (38.1)	74 (56.1)	—

Small bowel lesions that were considered to be the cause of the OGIB, such as angioectasia, dieulafoy's lesions, varices, arteriovenous malformations, ulcerations, multiple (≥3) erosions, diverticula, or the presence of blood and/or blood clots in the lumen of the small bowel, were considered as highly relevant lesions.

*P* values were calculated using the chi-squared test.

**Table 3:** Comparison of the CE findings between the HD and non-HD patients.

	HD patients (*N* = 19)	Non-HD patients (*N* = 23)	*P* value
Abnormal CE findings (highly relevant lesions)	11 (57.9)	15 (65.2)	0.88
Vascular lesions	10 (52.6)	10 (43.5)	0.55
Angioectasia	8 (42.1)	8 (34.8)	0.63
Arteriovenous malformations	0 (0)	1 (4.3)	—
Varices	0 (0)	1 (4.3)	—
Active bleeding with no identifiable cause	2 (10.5)	0 (0)	—
Erosive/ulcerated lesions	5 (26.3)	8 (34.8)	0.83
Ulcerations	2 (10.5)	4 (17.4)	0.89
Erosions (≥3)	3 (15.8)	4 (17.4)	0.93
No findings (less relevant lesions or no abnormalities)	8 (42.1)	8 (34.8)	—

Small bowel lesions that were considered to be the cause of the OGIB, such as angioectasia, dieulafoy's lesions, varices, arteriovenous malformations, ulcerations, multiple (≥3) erosions, diverticula, or the presence of blood and/or blood clots in the lumen of the small bowel, were considered as highly relevant lesions.

*P* values were calculated using the chi-squared test.

**Table 4:** Univariate and multivariate analyses to identify predictive factors for the presence of small bowel lesions in the CKD patients.

Variables	Vascular lesions				Erosive/ulcerated lesions			
	Univariate (OR 95% CI)	*P* value	Multivariate (OR 95% CI)	*P* value	Univariate (OR 95% CI)	*P* value	Multivariate (OR 95% CI)	*P* value
Age > 70 y	1.80 (0.53–6.14)	0.35			2.08 (0.55–7.79)	0.28		
Male sex	1.04 (0.30–3.57)	0.95			0.56 (0.15–2.04)	0.38		
Overt bleeding	7.00 (1.73–28.3)	0.006	2.73 (0.55–13.7)	0.22	0.65 (0.18–2.36)	0.51		
Blood transfusion	6.22 (1.63–23.8)	0.008	5.66 (1.10–29.1)	0.04	0.30 (0.75–1.19)	0.09		
History of maintenance HD	1.44 (0.43–4.90)	0.56			0.87 (0.24–3.15)	0.83		
Drinking	5.18 (1.15–23.3)	0.03	5.35 (0.86–33.3)	0.07	0.58 (0.13–2.59)	0.47		
Smoking	3.26 (0.90–11.1)	0.07			1.16 (0.32–4.26)	0.82		
Comorbidity								
Hypertension	0.90 (0.16–5.04)	0.90			1.00 (0.16–6.26)	>0.99		
Diabetes	1.18 (0.35–4.02)	0.79			1.00 (0.27–3.66)	>0.99		
Coronary artery disease	0.56 (0.16–1.89)	0.35			0.75 (0.21–2.73)	0.66		
Cerebral infarction	1.12 (0.20–6.30)	0.90			1.00 (0.16–6.26)	>0.99		
Liver cirrhosis	1.11 (0.07–18.9)	0.95			—	—		
Medication history								
Warfarin	2.01 (0.47–9.15)	0.34			0.23 (0.03–2.10)	0.19		
LDA	0.57 (0.16–2.01)	0.38			6.00 (1.13–31.9)	0.04	6.00 (1.13–31.9)	0.04
Thienopyridine	1.12 (0.20–6.30)	0.90			2.27 (0.40–13.1)	0.36		
NSAIDs	1.11 (0.07–18.9)	0.95			2.08 (0.12–35.9)	0.62		
H2-blockers	0.58 (0.15–2.21)	0.43			0.49 (0.11–2.18)	0.35		
PPIs	0.48 (0.13–1.81)	0.28			1.88 (0.49–7.15)	0.36		
Rebamipide	0.90 (0.22–3.53)	0.87			0.35 (0.07–1.92)	0.23		

CKD: chronic kidney disease; OR: odds ratio; CI: confidence interval; BMI: body mass index; HD: hemodialysis; LDA: low-dose aspirin; NSAIDs: nonsteroidal anti-inflammatory drugs; H2-blockers: histamine H2 receptor antagonists; PPIs: proton pump inhibitors.
